# Cluster analysis of 100 Marfan patients based on aortic 4D flow MRI and Z-score: insights into disease heterogeneity and stratification of subgroups

**DOI:** 10.1007/s00330-024-11034-6

**Published:** 2024-09-16

**Authors:** Alexander Lenz, Flora Bahr, Christoph Riedel, Felicia Wright, Martin Sinn, Shuo Zhang, Marion Schuett, Lennart Well, Gerhard Adam, Yskert von Kodolitsch, Bjoern P. Schoennagel, Peter Bannas

**Affiliations:** 1 Department of Diagnostic and Interventional Radiology and Nuclear Medicine, University Medical Center Hamburg-Eppendorf, Hamburg, Germany; 2 Clinical Science Department, Imaging Systems, Philips GmbH Market DACH, Hamburg, Germany; 3 Institute of Medical Biometry and Epidemiology, University Medical Center Hamburg-Eppendorf, Hamburg, Germany; 4 Department of Cardiovascular Medicine, University Heart and Vascular Center Hamburg, Hamburg, Germany

**Keywords:** 4D flow MRI, Marfan syndrome, Congenital heart disease, Aneurysm, Thoracic aorta

## Abstract

**Objectives:**

4D flow MRI-derived variables from Marfan patients are highly heterogeneous. Our aim was to identify distinct Marfan patient subgroups based on aortic 4D flow MRI and Z-score for stratification of distinct hemodynamic profiles and clinical features by means of hierarchical cluster analysis.

**Materials and methods:**

One hundred Marfan patients underwent baseline aortic 4D flow MRI at 3 T. Z-scores, degree of helical and vortical flow, wall shear stress, flow displacement, and peak velocity were determined in the ascending aorta. Sex, age, BMI, antihypertensive medication, and dural ectasia were recorded. Hierarchical cluster analysis was performed using 4D flow MRI variables and Z-scores as input.

**Results:**

Cluster analysis resulted in three distinct clusters characterized by different Z-scores (mean ± SD); cluster 1: 0.4 ± 1.1 vs. cluster 2: 3.1 ± 1.1 vs. cluster 3: 3.6 ± 1.9. The three clusters delivered differences in helical and vortical flow patterns (global *p* = 0.003 and *p* < 0.001, respectively), wall shear stress (0.49 ± 0.11 vs. 0.44 ± 0.12 vs. 0.37 ± 0.09 N/m^2^, global *p* < 0.001), flow displacement (0.11 ± 0.05 vs. 0.16 ± 0.08 vs. 0.15 ± 0.07, global *p* = 0.006), and peak velocity (76.3 ± 9.0 vs. 60.1 ± 7.3 vs. 56.0 ± 7.8 cm/s, global *p* < 0.001). Patients in cluster 1 and 2 were relevantly younger than in cluster 3 (32.3 ± 13.8 vs. 32.8 ± 12.6 vs. 40.2 ± 15.0 years, all pairwise ∆*p* < 0.0297).

**Conclusion:**

Hierarchical cluster analysis based on aortic 4D flow MRI and Z-score revealed three distinct subgroups of Marfan patients, each characterized by specific hemodynamic profiles and clinical features. Follow-up of our patients is warranted to assess if 4D flow MRI- and Z-score-based stratification can predict future aortic diameter growth and ultimately improve outcomes.

**Clinical relevance statement:**

A combination of Z-score and 4D flow MRI-derived parameters may help identify subgroups of Marfan patients representing different stages or phenotypes of aortic disease, which require specific management strategies.

**Key Points:**

- *Four-dimensional (4D) flow MRI-derived variables of Marfan patients are highly heterogeneous across varying Z-scores*.
- *Cluster analysis based on 4D flow MRI and Z-score revealed three distinct subgroups of Marfan patients*.
- *A combination of Z-score and 4D flow MRI-derived parameters may identify different stages of aortic disease in Marfan patients*.

**Graphical Abstract:**

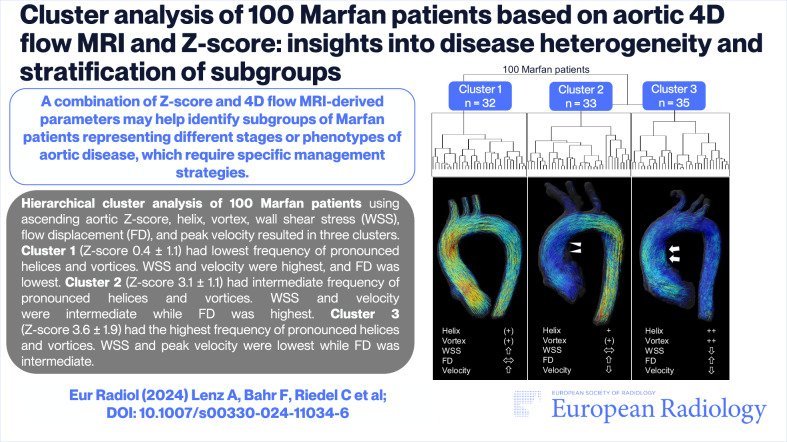

## Introduction

Marfan syndrome (MFS) is a potentially life-threatening disease with a prevalence of 1.5 to 17.2 per 100,000 individuals [1, 2]. The underlying mutation in the fibrillin-1 (FBN1) gene impairs blood vessel elasticity and results in progressive dilatation of the aorta [3]. Potentially fatal complications include aortic dissection and rupture [1].

Life-long annual aortic imaging is mandatory to determine the timepoint of aortic root replacement in Marfan patients [3, 4]. Unfortunately, the predictive value of aortic diameter measurements and Z-scores regarding adverse aortic events is limited [5, 6]. This underlines the need to identify new hemodynamic parameters as predictors for aortic diameter growth and adverse aortic events [7].

4D flow magnetic resonance imaging (MRI) has emerged as a promising technique for noninvasive assessment of aortic hemodynamics [7–13]. 4D flow MRI allows for the visualization and quantification of abnormal hemodynamic flow patterns such as *helical* and *vortical* flow. Moreover, it enables the quantification of other hemodynamic parameters, such as *wall shear stress* and *flow displacement* [7]. Previous studies have demonstrated significant differences between 4D flow MRI-derived variables of Marfan patients and healthy controls [14–19]. However, it is important to note that the 4D flow MRI-derived variables obtained from Marfan patients were highly heterogeneous. While certain studies have recognized the presence of heterogeneity within the Marfan population, this aspect was not a primary emphasis [18]. It is also worth noting that previous studies often had smaller cohorts, where the full extent of heterogeneity may not have been as evident. As a result, the nuances and variations within the Marfan population may have been overlooked. Therefore, further dedicated subgroup analysis is required to identify distinct subgroups of Marfan patients based on their 4D flow MRI-derived variables.

Hierarchical cluster analysis is a statistical method that aims to group similar individuals or objects based on multiple variables [20, 21]. In the context of Marfan syndrome, cluster analysis may help identify distinct subgroups of patients. We hypothesized that such subgroups represent different stages or phenotypes of aortic disease, which may have different outcomes and require tailored management strategies.

Therefore, the aim of this study was to apply hierarchical cluster analysis to identify distinct Marfan patient subgroups based on aortic 4D flow MRI and Z-score for stratification of hemodynamic profiles and clinical features.

## Materials and methods

### Study population

This prospective exploratory study was approved by the local ethics committee. Written informed consent was obtained from all patients.

Patients with a confirmed diagnosis of Marfan syndrome (MFS) according to the revised Ghent criteria who underwent 4D flow MRI between January 2017 and March 2021 at our institution were included in the study [22]. Diagnostic confirmation of MFS required the presence of a likely-pathogenic or pathogenic *FBN1* variant (Mutation in *FBN1* gene) [23]. All patients underwent 4D flow MRI, transthoracic echocardiography, ECG, and physical examination on the same day. Exclusion criteria were aortic root replacement, contraindications for MRI, age younger than 18 years, and pregnancy.

### Magnetic resonance imaging

Image acquisition was performed on a 3 Tesla system (Ingenia, Philips) with a 32-channel torso phased-array coil.

Free-breathing respiratory-gated and retrospectively cardiac-gated 4D flow MRI with full volumetric coverage of the thoracic aorta was acquired in para-sagittal orientation over the entire cardiac cycle. No contrast agent was administered. Scan parameters for 4D flow MRI included: acquired temporal resolution 24–38 ms, acquired spatial resolution 2.5 × 2.5 × 2.5 mm^3^, field of view (280–330) × (280–330) × [50–66] mm^3^, flip angle = 8°. Parallel imaging = SENSE (acceleration factor 4). A fixed velocity encoding value of 200 cm/s was chosen for streamlined clinical workflow. Scan time varied between 10 and 15 min depending on heart rate, respiratory pattern, and efficiency of respiratory gating.

All patients underwent ECG-gated balanced steady-state free precession (bSSFP) imaging to assess thoracic aortic diameters. bSSFP imaging with sensitivity encoding was acquired in the transversal and coronal plane as well as in para-sagittal orientation aligned with the curvature of the aortic arch [4, 24, 25]. Image acquisition was triggered to the end-diastolic phase of the cardiac cycle during end-expiratory breath-hold. Image parameters of the para-sagittal acquisition were as follows: TR/TE 2.4/0.98 ms; flip angle 60°; field of view 280 mm (FH) × 362 mm (AP) × 113 mm (RL); matrix, 282 × 216; number of slices 20; acquired voxel size 1.3 × 1.3 × 9 mm, SENSE factor 4. Acquisition time for each stack 11–15 s (depending on the individual heart rate) [25].

All patients underwent sagittal T2-weighted MRI of the lumbar spine to assess the presence of dural ectasia according to Oosterhof et al as part of the imaging routine [26].

### Calculation of Z-scores

Measurements of the ascending aorta at the level of the bulbus aortae were performed perpendicular to the blood-filled lumen in para-sagittal orientation using inner-to-inner edge diameters. Using the identically oriented para-sagittal images avoided user influence introduced by individually performed MPRs [25, 27]. Z-scores for each Marfan patient were calculated based on aortic diameter, body surface area, and age [28, 29]. Z-scores express the deviation from a normative size- or age-specific population mean. A Z-score between −2 and +2 is considered normal [30].

### 4D flow MRI data analysis

4D flow MRI data were corrected for background phase offsets and phase aliasing [31]. All data sets were automatically reconstructed to 24 time frames per cardiac cycle and three-dimensional angiograms were rendered using dedicated software (GTFlow 3.2.4, GyroTools LLC).

One radiologist (A.L.) with 5 years of experience in 4D flow MRI assessment manually placed analysis planes at three defined anatomic landmarks in the thoracic aorta: (1) sinotubular junction (STJ), (2) mid-ascending aorta (midAA), and (3) aortic arch proximal to the brachiocephalic trunk (proxAA) [24].

Two radiologists with 5 years (A.L.) and 2 years (F.B.) experience in 4D flow MRI independently evaluated *helical* and *vortical* blood flow patterns in the AA at systole according to a 3-point scale: 0 (none), 1 (< 360°, moderate), and 2 (> 360°, pronounced). A *helical* flow pattern was defined as a regional spiral movement along the blood flow direction. A *vortical* flow pattern was defined as a localized re-circulating movement with flow deviating from the physiological flow direction by > 90° [18, 32]. In case of discordant readings, a third radiologist with 5 years of experience (P.B.) stepped in to reach a consensus.

Magnitudinal *wall shear stress* was derived from each analysis plane at peak systole [9, 33]. Values for were averaged per slice over five cardiac time frames centered on peak systole to reduce measurement noise [18, 34].

*Flow displacement* was semiautomatically quantified and normalized for local diameter using a custom-built MATLAB script (MATLAB R2021b, The MathWorks, Inc.), as reported previously by Sigovan et al and Mahadevia et al [34–36].

*Peak velocity* was derived from each analysis plane at peak systole.

### Statistical analysis

All data were summarized using descriptive statistics. Categorical data are summarized by absolute and relative frequencies. A linearly weighted κ test was used to assess the inter-observer agreement of blood flow pattern analyses. Continuous data are summarized by mean, standard deviation, median, first and third quartile, minimum and maximum.

To investigate differences between groups of Z-scores, unpaired *t*-test or ANOVA, as appropriate, was used for continuous variables, and Pearson’s chi-squared test or Fisher’s exact test, as appropriate, for categorical variables. In this exploratory data analysis, all variables were compared and statistically assessed for descriptive purposes and not in a confirmatory sense. Therefore, *p*-values are not adjusted for multiple testing but are used as descriptive measures. All *p*-values < 0.05 were considered statistically significant.

*Wall shear stress*, *flow displacement* and *peak velocity* were analyzed regarding a correlation with Z-score using Pearson’s correlation coefficient *r*. The strength of the linear relationship corresponding to the correlation coefficient value was defined as strong when ≥ 0.6 [37].

No missing data methods needed to be applied, as all data was collected.

All analyses were conducted using R version 4.3.0 (R Foundation for Statistical Computing).

### Cluster analysis

Hierarchical cluster analysis was applied for the identification of distinct Marfan patient subgroups. The variables Z-score, *helix*, and *vortex* in the AA, as well as *wall shear stress*, *flow displacement*, and *peak velocity* at STJ were used for the cluster analysis.

All variables were standardized using the z-transformation (mean = 0, std = 1). The Euclidian distance was calculated as the distance measure between all patients using the agglomerative, hierarchical Ward cluster method [38]. First, one separate cluster was assigned to every data point. Then two nearest clusters were combined into bigger and bigger clusters recursively until there was only one single cluster left. Second, to discuss and define the number of clusters, the Euclidian distance over normalized values was used to calculate the average silhouette coefficient (a metric for evaluating the clustering performance) [39]. Finally, the hierarchical method was compared to the non-hierarchical k-means method [40].

## Results

### Study population

4D flow MRI was successfully performed on 100 Marfan patients. Mean age was 35.2 ± 14.2 years, and 69% of patients were female. The mean diameter of the bulbus aortae was 38.5 ± 5.8 mm, with a mean Z-score of 2.4 ± 2.0. Detailed patient characteristics are reported in Table 1.

**Table 1:** Patient characteristics and 4D flow MRI-derived parameters of 100 Marfan patients

Characteristic	All patients *n* = 100
Sex, female	69 (69%)
Age (years)	35.2 ± 14.2
Diameter bulbus aortae (mm)	38.5 ± 5.8
Height (cm)	184.1 ± 10.6
Weight (kg)	79.5 ± 19.4
BMI	23.3 ± 4.8
Z-score	2.4 ± 2.0
BP syst (mmHg)	126.3 ± 14.5
BP diast (mmHg)	75.6 ± 9.3
Antihypertensive medication, yes	44 (44%)
Dural ectasia, yes	33 (33%)
Helix (grade)	
0	34 (34%)
1	45 (45%)
2	21 (21%)
Vortex (grade)	
0	56 (56%)
1	31 (31%)
2	13 (13%)
Wall shear stress mag (N/m2)	
STJ	0.43 ± 0.12
midAA	0.57 ± 0.13
proxAA	0.55 ± 0.13
Flow displacement	
STJ	0.14 ± 0.07
midAA	0.25 ± 0.08
proxAA	0.25 ± 0.08
Peak velocity (cm/s)	
STJ	63.9 ± 11.8
midAA	59.5 ± 12.3
proxAA	65.3 ± 13.4

Values represent mean ± SD or numbers and percentages

*BP* blood pressure, *STJ* sinotubular junction, *midAA* mid-ascending aorta, *proxAA* proximal aortic arch

### 4D flow MRI-derived qualitative blood flow alterations according to Z-score

4D flow MRI allowed visualization of aortic flow alterations, such as regional *helical* and *vortical* flow patterns in the AA.

There was substantial agreement between the two readers on grading of *helical* flow (κ = 0.78, 95% CI from 0.627 to 0.852), and almost perfect agreement on grading of *vortical* flow (κ = 0.85, CI from 0.703 to 0.913).

A total of 21 patients showed pronounced *helical* flow (grade 2), 45 patients moderate helical flow (grade 1), and 34 patients no *helical* flow (grade 0) in the AA.

A total of 13 patients showed pronounced *vortical* flow (grade 2), 31 patients moderate vortical flow (grade 1), and 56 patients no *vortical* flow (grade 0) in the AA (Table 1).

Patients with moderate (grade 1) and pronounced (grade 2) *helical* flow revealed higher Z-scores when compared to patients with physiological flow (grade 0) (mean ± SD: grade 0 = 1.96 ± 1.71 vs. grade 1 = 2.56 ± 1.85 vs. grade 2 = 2.81 ± 2.55, global *p* = 0.24).

**Fig. 1:**
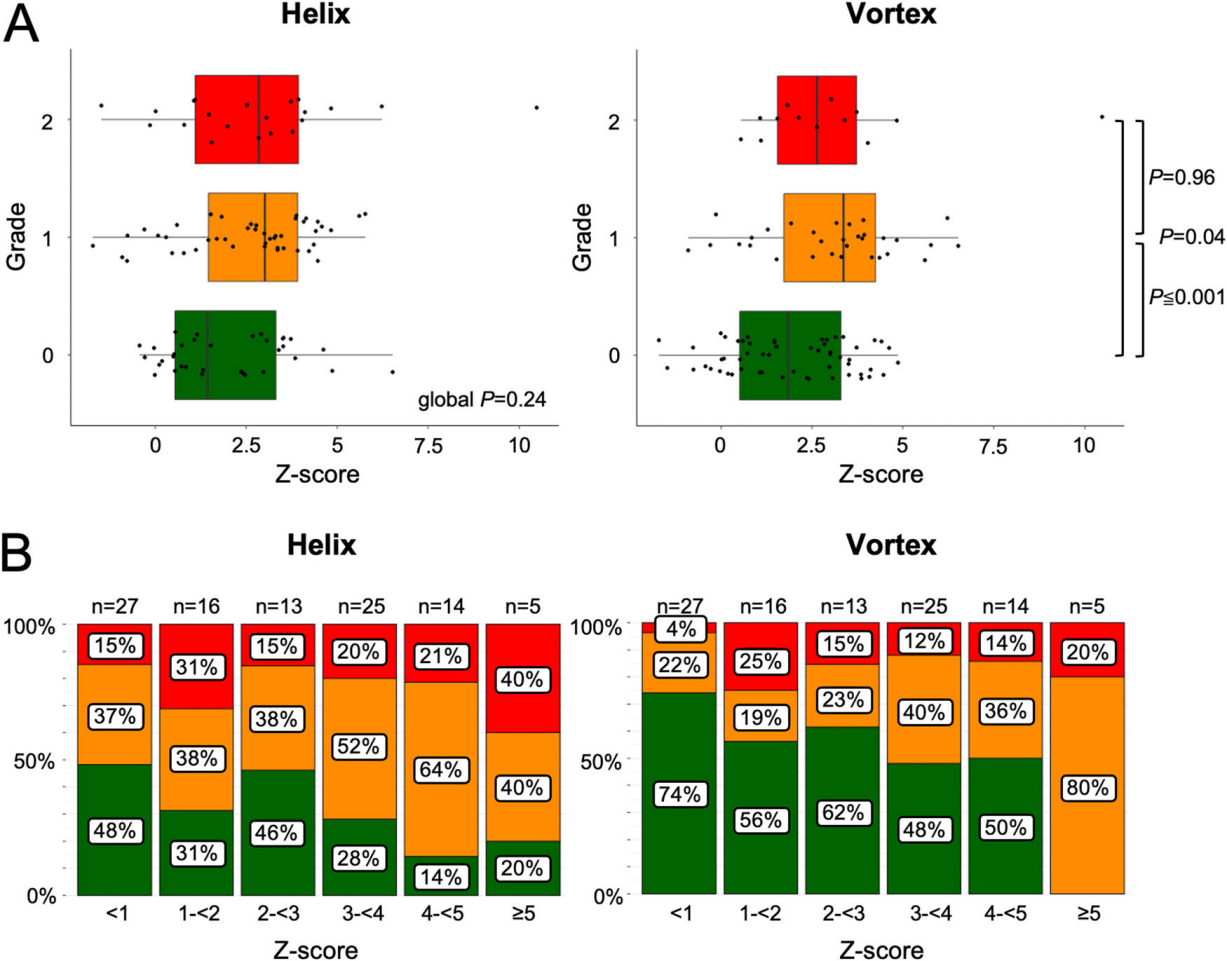
Distribution of 4D flow MRI-derived *helical* and *vortical* blood flow in the ascending aorta according to Z-scores in 100 Marfan patients. **A** Marfan patients with moderate (grade 1) and pronounced (grade 2) *helical* and *vortical* blood flow had higher Z-scores compared to those with physiological flow (grade 0). Global *p*-values assessed by one-way ANOVA and pairwise testing, if applicable. **B** Distribution of physiological (green), moderate (yellow), and pronounced (red) flow patterns across Z-scores, showing an increase in moderate and pronounced flows with increasing Z-scores

Patients with moderate (grade 1) and pronounced (grade 2) *vortical* flow revealed higher Z-scores when compared to patients with physiological blood flow (grade 0) (grade 0 = 1.88 ± 1.72 vs. grade 1 = 3.07 ± 1.92 vs. grade 2 = 3.11 ± 2.56; grade 1 vs. grade 0: ∆ = 1.19 *p* = 0.0064, grade 2 vs. grade 0: ∆ = 1.22 *p* = 0.04, grade 1 vs. grade 2: ∆ = 0.03 *p* = 0.96) (Fig. 1A).

Overall frequencies of both moderate and pronounced flow patterns increased with increasing Z-scores (Fig. 1B). Of note, all patients with Z-scores ≥ 5 revealed *vortical* flow patterns.

### Correlation of 4D flow MRI-derived quantitative blood flow parameters and Z-score

**Fig. 2:**
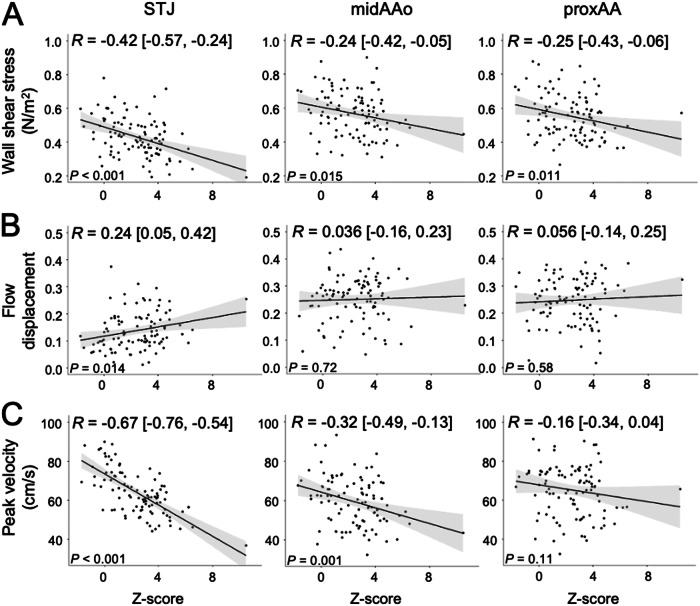
Correlation of 4D flow MRI-derived *wall shear stress*, *flow displacement*, and *peak velocity* with Z-scores in the ascending aorta of 100 Marfan patients. **A** *Wall shear stress* revealed a moderate negative correlation with Z-scores at all three aortic levels. **B** *Flow displacement* revealed a mild correlation with Z-scores at the sinotubular junction (STJ) and no correlation at the mid-ascending aorta (midAA) and proximal aortic arch (proxAA). **C** *Peak velocity* revealed a strong negative correlation with Z-scores at the STJ and a moderate negative correlation at the midAA. Pearson’s correlation coefficients (R) are reported with 95% confidence intervals in parentheses

Wall shear stress revealed a moderate negative correlation with Z-scores at STJ (*r* = −0.42), and a weak correlation at midAA and proxAA (*r* = −0.24 and *r* = −0.25, respectively) (Fig. 2A).

*Flow displacement* revealed a weak correlation with Z-scores at STJ (*r* = 0.24) and no correlation at midAA and proxAA (*r* = 0.036 and *r* = 0.056, respectively) (Fig. 2B).

*Peak velocity* revealed a strong negative correlation with Z-scores at STJ (*r* = −0.67) and a moderate negative correlation at midAA (*r* = −0.32) and no correlation at proxAA (*r* = −0.16) (Fig. 2C).

### 4D flow MRI-derived blood flow variables in Marfan patients with Z-scores < 2 and ≥ 2

Of the 100 Marfan patients, 43 patients had a Z-score < 2, while 57 patients had an elevated Z-score ≥ 2.

**Fig. 3:**
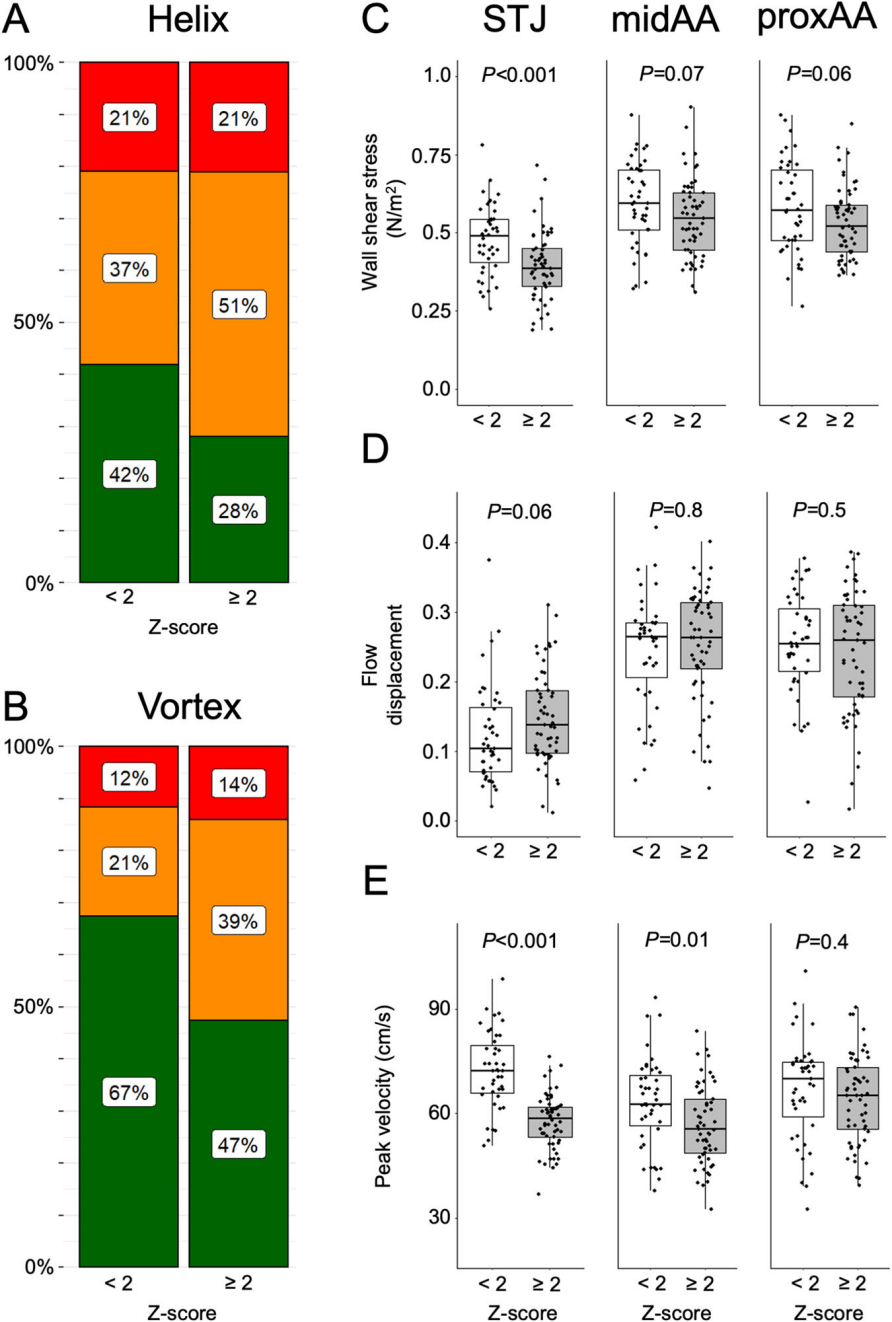
Frequencies of 4D flow MRI-derived *helical* and *vortical* blood flow and comparison of *wall shear stress*, *flow displacement*, and *peak velocity* in the ascending aorta according to Z-scores < 2 and ≥ 2. Of the 100 Marfan patients, 43 patients had a Z-score < 2, while 57 patients had a Z-score ≥ 2. Frequencies of both moderate (yellow) and pronounced (red) *helical* (**A**) and *vortical* (**B**) flow patterns combined are higher in Marfan patients with Z-scores ≥ 2, without reaching a significant difference (all *p* > 0.1). **C** Patients with Z-scores ≥ 2 showed lower *wall shear stress* at STJ, with no relevant reduction at midAA and proxAA. **D** *Flow displacement* was higher across all levels for patients with Z-scores ≥ 2. **E** *Peak velocity* was decreased in patients with Z-scores ≥ 2 at STJ and midAA, with no relevant decrease at proxAA

Both moderate and pronounced *helical* and *vortical* flow patterns combined were more frequent in patients with Z-scores ≥ 2 than in patients with Z-scores < 2, however, without reaching a significant difference (all *p* > 0.1) (Fig. 3, Table 2).

**Table 2:** Patient characteristics and 4D flow MRI-derived parameters of 100 Marfan patients according to Z-score < 2 and ≥ 2

Variable	Z-score < 2 (*n* = 43)	Z-score ≥ 2 (*n* = 57)	*p*-value
Sex, female	28 (65%)	41 (72%)	0.5
Age (years)	36.9 ± 15.5	33.9 ± 13.1	0.3
Diameter bulbus aortae (mm)	34.0 ± 0.4	41.9 ± 0.5	**< 0.001**
BMI	24.3 ± 5.7	22.6 ± 4.0	0.07
Z-score	0.56 ± 0.9	3.81 ± 1.3	**< 0.001**
Antihypertensive medication, yes	24 (56%)	20 (35%)	0.04
Dural ectasia, yes	20 (47%)	13 (23%)	**0.01**
Helix (grade)			0.3
0	18 (42%)	16 (28%)	
1	16 (37%)	29 (51%)	
2	9 (21%)	12 (21%)	
Vortex (grade)			0.11
0	29 (67%)	27 (47%)	
1	9 (21%)	22 (39%)	
2	5 (12%)	8 (14%)	
Wall shear stress mag (N/m2)			
STJ	0.48 ± 0.11	0.39 ± 0.11	**< 0.001**
midAA	0.59 ± 0.13	0.55 ± 0.12	0.07
proxAA	0.58 ± 0.15	0.53 ± 0.11	0.06
Flow displacement			
STJ	0.12 ± 0.07	0.15 ± 0.07	0.06
midAA	0.25 ± 0.08	0.25 ± 0.08	0.8
proxAA	0.25 ± 0.07	0.24 ± 0.09	0.5
Peak velocity (cm/s)			
STJ	72.1 ± 11.2	57.7 ± 7.8	**< 0.001**
midAA	63.1 ± 12.8	56.7 ± 11.2	**0.01**
proxAA	66.7 ± 14.6	64.3 ± 12.5	0.4

Statistically significant *p*-values are in bold

Values represent mean ± SD or numbers and percentages. *p*-values: Pearson’s chi-squared test or two-sample *t*-test was used

*STJ* sinotubular junction, *midAA* mid-ascending aorta, *proxAA* proximal aortic arch

*Wall shear stress* was significantly lower in patients with Z-scores ≥ 2 compared to Z-scores < 2 at STJ (*p* < 0.001). At midAA and proxAA *wall shear stress* was reduced, however, without reaching a significant difference (all *p* > 0.06) (Fig. 3C, Table 2).

*Flow displacement* was similar in patients with Z-scores ≥ 2 compared to < 2 at all three levels (all *p* > 0.06) (Fig. 3D, Table 2).

*Peak velocity* was significantly lower in patients with Z-scores ≥ 2 at STJ and midAA (*p* < 0.001 and *p* < 0.01, respectively), with no difference at proxAA (*p* = 0.4) (Fig. 3E, Table 2).

### Hierarchical cluster analysis according to Z-scores and 4D flow MRI-derived variables

The hierarchical cluster analysis in the 100 Marfan patients resulted in two, three and four clusters with an average silhouette width of 0.22, 0.18, and 0.16, respectively.

Two clusters had the highest silhouette width but appeared to segregate primarily based on Z-score differences. Selecting three clusters for our analysis provided an optimal balance between capturing the essential heterogeneity of the condition and maintaining a clinically relevant and interpretable framework.

**Fig. 4:**
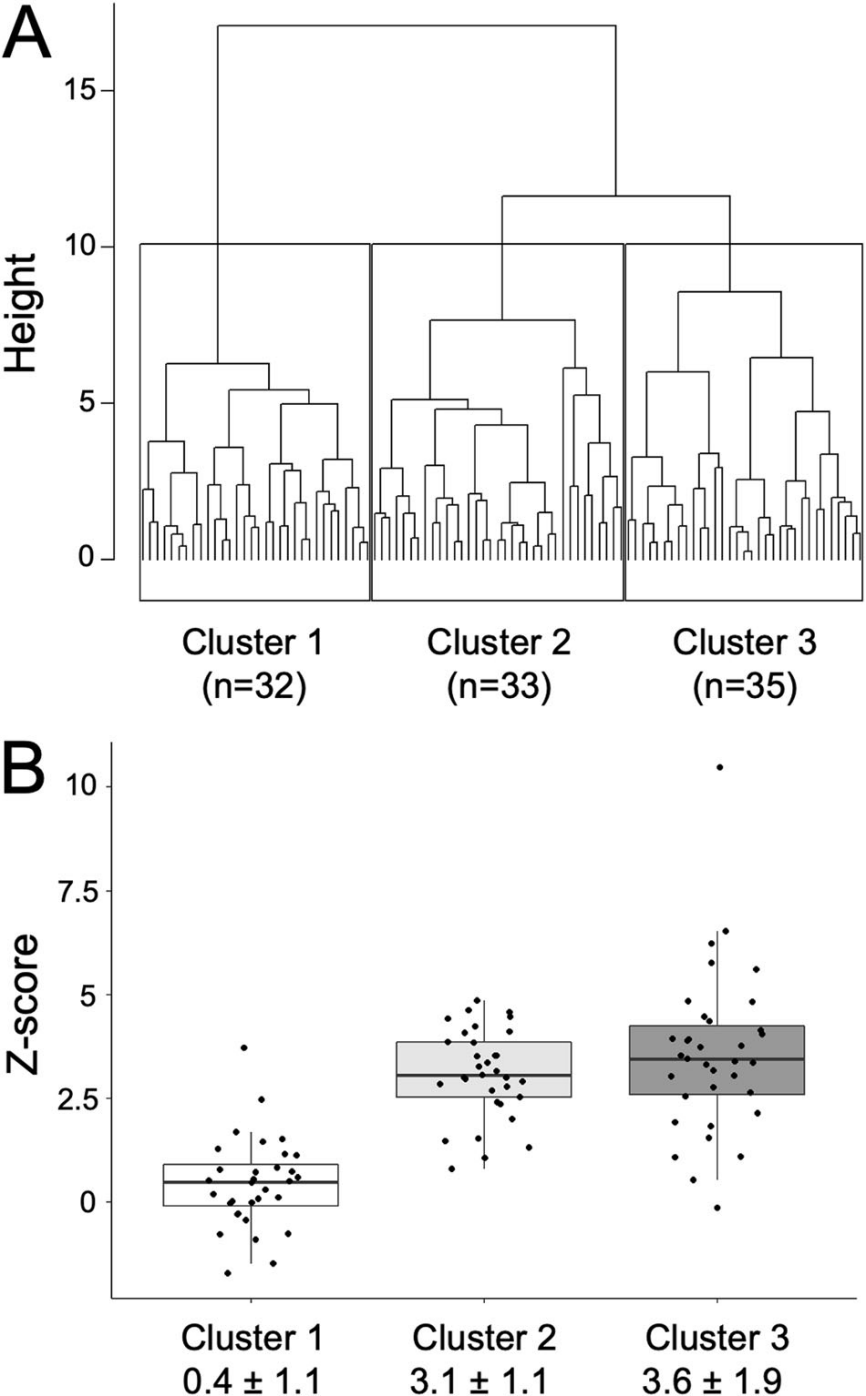
Hierarchical cluster analysis of 100 Marfan patients according to Z-scores and 4D Flow MRI-derived parameters. We included six 4D flow MRI-derived variables: Z-score, *helix*, *vortex*, *wall shear stress*, *flow displacement*, and *peak velocity*. The hierarchical cluster analysis resulted in three groups with an optimized balance between group size and heterogeneity. **A** The resulting cluster dendrogram illustrates the increasing heterogeneity of the clusters, indicated by the Euclidean distance. Numbers in parentheses indicate the number of Marfan patients in each cluster. **B** Box-plot analysis of the distribution of Z-scores in the three clusters. Numbers indicate mean ± SD

The three clusters (C) had similar size: C1 (*n* = 32), C2 (*n* = 33) and C3 (*n* = 35) (Table 3). The dendrogram of the hierarchical clustering is shown in Fig. 4A.

**Table 3:** Results of hierarchical cluster analysis of 100 Marfan patients according to Z-scores and 4D flow CMR-derived parameters

Variables used to form the clusters	Cluster 1 (*n* = 32)	Cluster 2 (*n* = 33)	Cluster 3 (*n* = 35)	*p*-value
Z-score	0.4 ± 1.1^a^	3.1 ± 1.1^b^	3.6 ± 1.9	**< 0.001**
Helix				**0.003**
0	16 (50.0%)	14 (42.4%)	4 (11.4%)	
1	14 (43.8%)	12 (36.4%)	19 (54.3%)	
2	2 (6.2%)	7 (21.2%)	12 (34.3%)	
Vortex				**< 0.001**
0	27 (84.4%)	29 (87.9%)	0 (0.0%)	
1	5 (15.6%)	4 (12.1%)	22 (62.9%)	
2	0 (0.0%)	0 (0.0%)	13 (37.1%)	
Wall shear stress STJ (N/m^2^)	0.49 ± 0.11	0.44 ± 0.12	0.37 ± 0.09	**< 0.001**^**e**^
Flow displacement STJ	0.11 ± 0.05^a^	0.16 ± 0.08	0.15 ± 0.07^b^	**0.006**
Peak velocity STJ (cm/s)	76.3 ± 9.0	60.1 ± 7.3	56.0 ± 7.8	**< 0.001**^**e**^
Other variables				
Sex				0.8
Male	10 (31.2%)	9 (27.3%)	12 (34.3%)	
Female	22 (68.8%)	24 (72.7%)	23 (65.7%)	
Age (years)	32.3 ± 13.8^c^	32.8 ± 12.6^c^	40.2 ± 15.0^d^	**0.036**
BMI	23.7 ± 4.8	23.1 ± 5.4	23.2 ± 4.2	0.9
Antihypertensive medication				0.4
Medication	17 (53.1%)	12 (36.4%)	15 (42.9%)	
No medication	15 (46.9%)	21 (63.6%)	20 (57.1%)	
Dural ectasia				0.2
Dural ectasia	13 (40.6%)	7 (21.2%)	13 (37.1%)	
No dural ectasia	19 (59.4%)	26 (78.8%)	22 (62.9%)	

Statistically significant *p*-values are in bold

The upper part of the table indicates the distribution of the variables that were used to form the three clusters, including Z-score, *helix*, *vortex*, *wall shear stress*, *flow displacement* and *peak velocity* derived from 4D flow MRI. The lower part of the table indicates the distribution of other variables given the three defined clusters. Numbers indicate mean ± SD. *p*-values: ANOVA was used for continuous variables and, as appropriate, Fisher’s exact test or Pearson’s chi-squared test for categorical variables

^a^Mean difference to cluster 3: *p* < 0.05

^b^Mean differences to cluster 1 and cluster 2: *p* < 0.05

^c^ Mean difference to cluster 2 and cluster 3: *p* < 0.05

^d^Mean difference to cluster 1: *p* < 0.05

^e^All pairwise mean differences: *p* < 0.05

Z-scores within the three clusters revealed one cluster with physiological Z-scores (C1: 0.4 ± 1.1) and two clusters with pathologically increased Z-scores (C2: 3.1 ± 1.1 and C3: 3.6 ± 1.9) (∆C2-C1 = 2.65 95% CI (1.94, 3.36) *p* < 0.0001, ∆C3-C1 = 3.12 95% CI (2.42, 3.82) *p* < 0.0001, ∆C3-C2 = 0.47 95% CI (−0.23, 1.16), *p* = 0.18) (Fig. 4B).

**Fig. 5:**
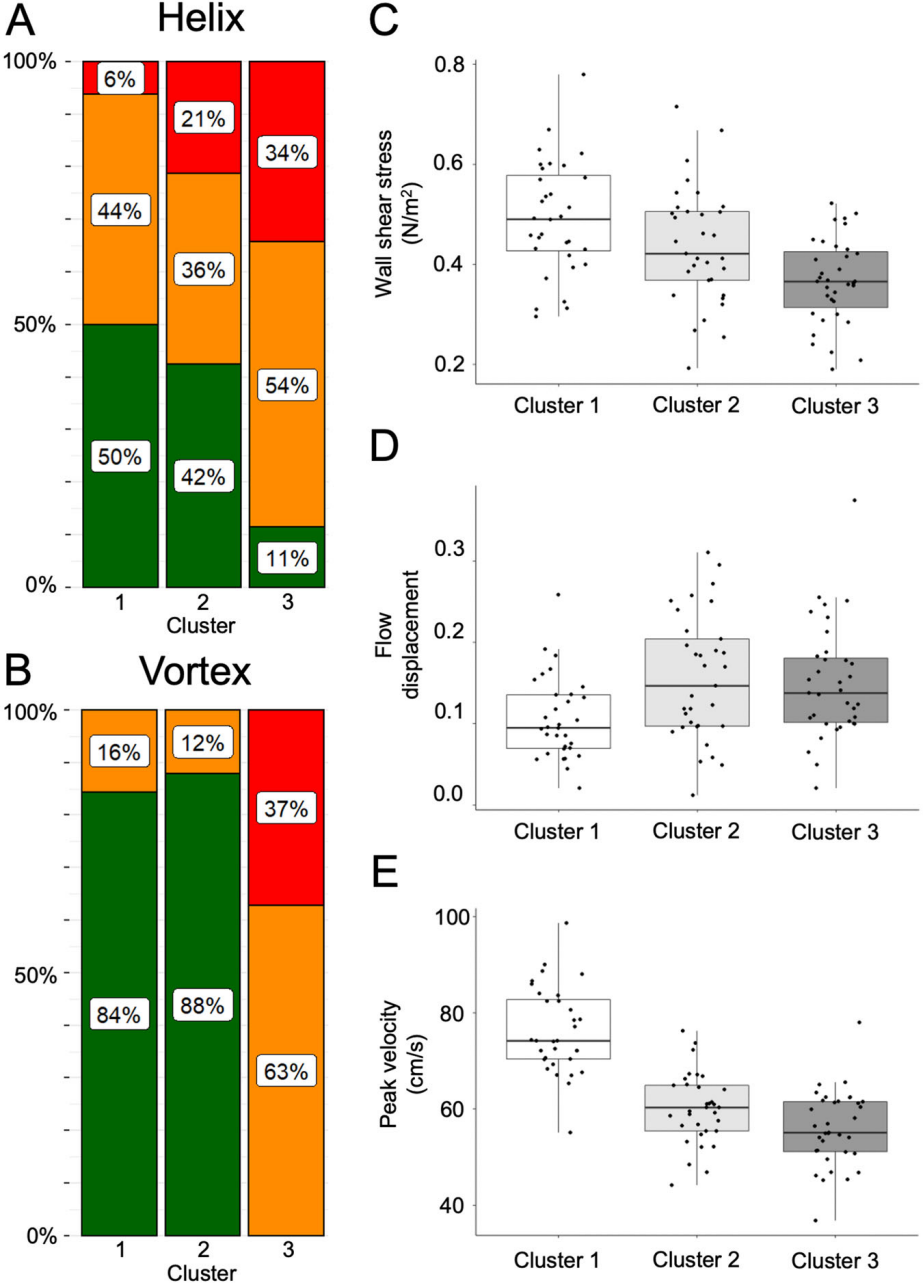
Frequencies of 4D flow MRI-derived *helical* and *vortical* blood flow patterns and comparison of *wall shear stress*, *flow displacement*, and *peak velocity* in the ascending aorta in the three identified clusters in 100 Marfan patients. **A** Note the increase of both moderate (yellow) and pronounced (red) *helical* flow in clusters 2 and 3, as compared to cluster 1. **B** Of note, no pronounced vortices were observed in clusters 1 and 2, while no physiological flow was observed in cluster 3. **C** *Wall shear stress* was highest in cluster 1, followed by cluster 2 and cluster 3 (all pairwise comparisons *p* < 0.037). **D** *Flow displacement* was highest in cluster 2, followed by cluster 3 and cluster 1 (∆Cluster 2-Cluster 1 and ∆Cluster 3-Cluster 1: *p* < 0.009). **E** *Peak velocity* was highest in cluster 1, followed by cluster 2 and cluster 3 (all pairwise comparisons *p* < 0.04)

**Fig. 6:**
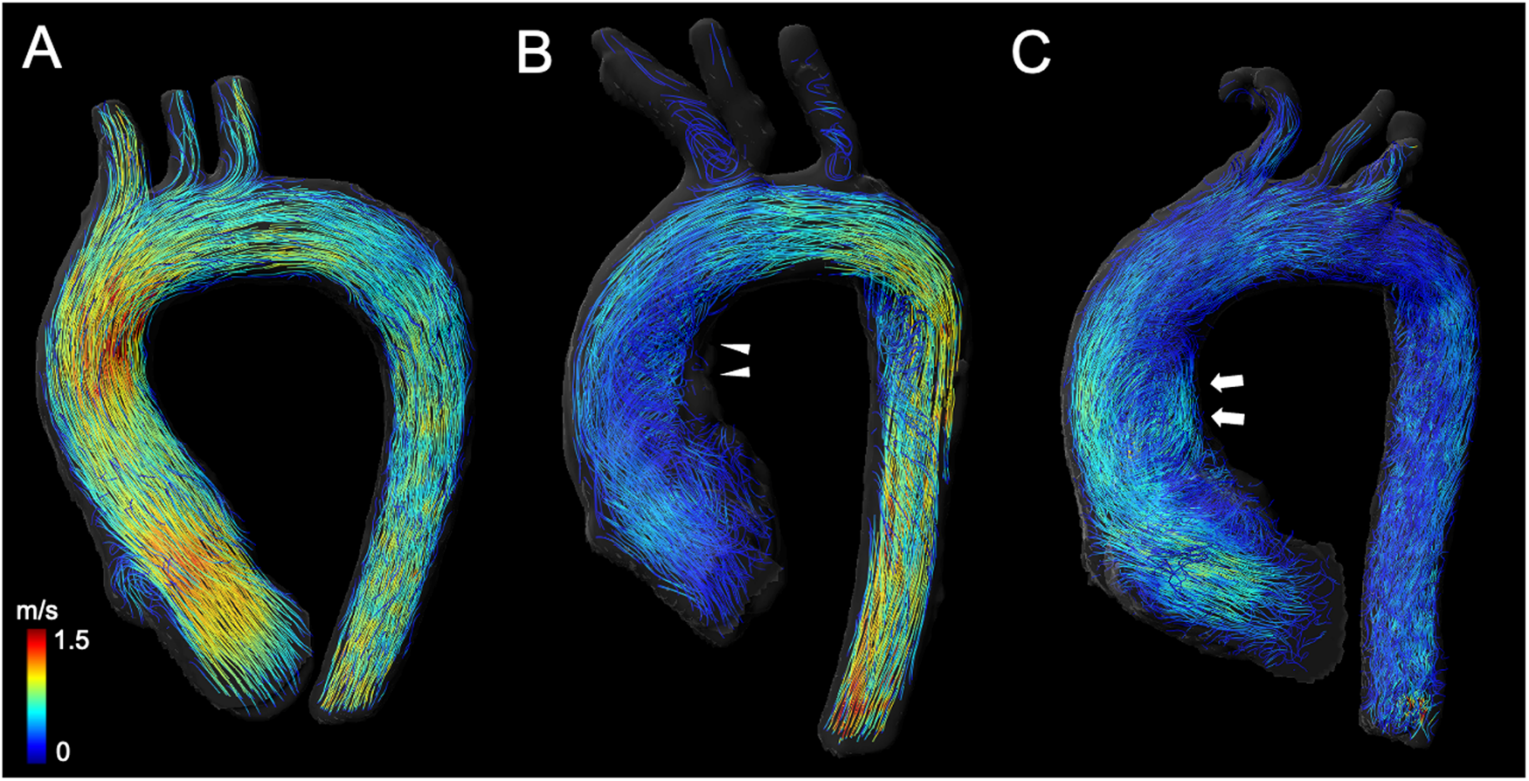
4D flow MRI-based streamline visualization of hemodynamics in the thoracic aorta of Marfan patients according to the three identified clusters. **A** Physiological blood flow in a 41-year-old female Marfan patient from cluster 1 with a Z-score of 0.3. **B** Moderate *helical* (grade 1) blood flow in a 20-year-old female Marfan patient from cluster 2 with a Z-score of 3.4. **C** Pronounced combined *helical* (grade 2) and *vortical* (grade 2) blood flow in a 60-year-old female Marfan patient from cluster 3 with a Z-score of 4.0

*Helical* flow patterns were most frequent in cluster 3, followed by cluster 2 and 1 (global *p* = 0.003) (Figs. 5A, 6, movie_cluster 1–3).

*Vortical* flow patterns were also most frequent in cluster 3, followed by cluster 2 and 1. Of note, no pronounced *vortices* were observed in clusters 1 and 2, while no physiological flow (0%) and all pronounced *vortices* (37%) were observed in cluster 3 (global *p* < 0.001) (Figs. 5B, 6, movie_cluster 1–3).

*Wall shear stress* at STJ was highest in cluster 1, followed by cluster 2 and 3 (all pairwise ∆*p* < 0.037) (Fig. 5C and Table 3).

*Flow displacement* at STJ was highest in cluster 2, followed by cluster 3 and 1 (∆C1-C2 and C1-C3 *p* < 0.009) (Fig. 5D and Table 3).

*Peak velocity* at STJ was highest in cluster 1, followed by cluster 2 and 3 (all pairwise ∆*p* < 0.04) (Fig. 5E and Table 3).

In terms of other variables, age was higher in cluster 3 when compared to cluster 1 and 2 (∆C3-C1 and C3-C2 *p* < 0.0297). Sex, BMI, antihypertensive medication, and dural ectasia showed no relevant differences between the three clusters (all global *p* > 0.2) (Table 3).

To check the robustness of the chosen hierarchical Ward cluster method, the results were compared to the non-hierarchical k-means method [40]. The k-means method delivered similar numbers of patients in three clusters. Both methods identified clusters with similar Z-score distributions (hierarchical: 0.8, 3.2, and 3.5 vs. k-means: 0.4, 3.1, and 3.6). However, hierarchical clustering offered clearer differentiation of 4D flow variables when compared to the k-means method.

## Discussion

Our study indicates that 4D flow MRI-derived hemodynamic variables are associated with Z-scores in Marfan patients in the ascending aorta. Using hierarchical clustering based on 4D flow MRI-derived variables and Z-scores, we were able to identify three subgroups of patients with distinct hemodynamic profiles and clinical features (Fig. 6 and movie_cluster 1–3).

Corresponding to the results of previous studies, we observed *helices* and *vortices* in the ascending aorta more frequently in Marfan patients with higher Z-scores [14, 17–19, 41, 42].

In our study, *wall shear stress* in the ascending aorta was negatively correlated with Z-scores. This finding is consistent with earlier findings, which have observed a correlation between decreased *wall shear stress* and increased aortic diameter in Marfan patients. This suggests that lower *wall shear stress* could signal abnormal vascular conditions in the aorta and serve as a potential marker for disease advancement [17, 18, 42].

Accordingly, *peak velocity* was also negatively correlated with Z-scores. *Flow displacement* was not correlated with Z-scores. This is explained by the rather symmetrical dilatation of the aortic root in Marfan patients and the patent aortic valve, as opposed to patients with bicuspid aortic valve disease, where *flow displacement* is primarily due to the asymmetry of the aortic valve [34].

Next, we compared 4D flow MRI-derived variables in patients with Z-scores < 2 and ≥ 2. Frequencies of both altered *helical* and *vortical* flow tended to be higher in patients with Z-scores ≥ 2. Interestingly, pronounced *vortical* and *helical* flow was observed in a subgroup of patients with non-pathologic Z-scores (< 2), while there was also a subgroup of patients with pathological Z-scores (≥ 2) without any pronounced flow patterns in the ascending aorta.

*Wall shear stress* and *peak velocities* were relevantly reduced in patients with Z-scores ≥ 2 at STJ. However, both variables were distributed heterogeneously across both groups.

*Flow displacement* showed no relevant difference between these two groups.

It is important, that our study in 100 Marfan patients indicates that 4D flow MRI-derived variables are highly heterogeneous across varying Z-scores. Our study extends the existing literature [14, 15, 17] by indicating that normal Z-scores are not necessarily associated with physiological flow profiles, while pathologically increased Z-scores are not necessarily associated with pronounced flow profiles.

There may be several potential explanations: First, 4D flow MRI might be sensitive enough to detect early hemodynamic changes in the aorta before structural abnormalities, such as dilatation, become evident. This early detection could be crucial for identifying patients at risk even before traditional measures such as the aortic diameter show significant changes.

Second, Marfan syndrome may exhibit regional variations in aortic wall properties. 4D flow MRI could be capturing alterations in hemodynamics related to localized changes in aortic stiffness, compliance, or other biomechanical factors that may precede global changes in aortic dimensions.

Third, Marfan syndrome is known for its genetic heterogeneity, and patients may exhibit diverse phenotypic expressions. The abnormal flow patterns in patients with normal aortic diameters could be related to genetic factors influencing aortic hemodynamics independently of aortic size.

Fourth, some patients may develop compensatory mechanisms that maintain normal flow despite elevated Z-scores. These mechanisms could include adaptive changes in the vascular architecture, or other hemodynamic adjustments that help mitigate the impact of aortic dilatation.

This observation may have important clinical implications. One might hypothesize that, on one hand, patients with normal Z-scores but pronounced flow profiles may have higher aortic growth rates and a higher risk for aortic events. On the other hand, patients with elevated Z-scores but physiological flow profiles may have lower growth rates. A combination of Z-score and 4D flow MRI-derived parameters may, therefore, identify different stages or phenotypes of aortic disease. Incorporating this information into current diagnostic algorithms may improve risk stratification and treatment decision-making for Marfan patients.

We therefore performed a hierarchical cluster analysis to group similar Marfan patients based on multiple 4D flow MRI-derived variables and Z-scores. Hierarchical cluster analysis using ascending aortic Z-score, *helix*, *vortex*, *wall shear stress*, *flow displacement*, and *peak velocity* resulted in three clusters of Marfan patients.

Cluster 1 had low physiological, Z-scores (0.4 ± 1.1) and the least *helical* flow and no pronounced *vortical* flow. *Wall shear stress* and *peak velocity* were highest, and *flow displacement* was lowest. Patients in cluster 1 had the lowest mean age (32 ± 14 years).

Cluster 2 had an elevated pathological Z-score (3.1 ± 1.1). Cluster 2 had intermediate frequency of *helical* flow and no pronounced *vortical* flow. *Wall shear stress* and *peak velocity* were intermediate. *Flow displacement* was highest. Patients in cluster 2 were also young, with a mean age of 33 ± 13 years.

Cluster 3 had the highest Z-score (3.6 ± 1.9) and most *helical* flow and all highly pronounced *vortical* flow. *Wall shear stress* and *peak velocity* were the lowest. *Flow displacement* was intermediate. Patients in cluster 3 had the highest mean age (40 ± 15 years).

Other variables, such as sex, BMI, and dural ectasia, were evenly distributed across the three clusters.

The identification of distinct patient subgroups based on hemodynamic profiles offers the opportunity for personalized risk stratification and treatment planning. It is tempting to speculate that patients in cluster 1 might be regarded at the lowest risk for future aortic dilatation and aortic events. As such, these patients may require less intensive monitoring and treatment compared to those in clusters 2 and 3. Conversely, patients in cluster 3, may be regarded at higher risk for accelerated aortic dilatation and aortic events. These patients may benefit from more frequent monitoring and aggressive management strategies to mitigate their heightened risk for aortic dissection or rupture. This personalized approach could lead to more targeted interventions, optimized resource allocation, and ultimately improved patient outcomes.

We will verify these hypotheses in a subsequent longitudinal study by follow-up of our patients to assess if our 4D flow MRI and Z-score-based stratification can predict future aortic diameter growth and ultimately improve patient outcomes by a more individualized approach to disease monitoring.

The main limitation of our current study is its cross-sectional nature.

Second, our study’s limitations include the inherent variability in cluster analysis outcomes, influenced by the choice of clustering methods, distance metrics, and cluster numbers. Furthermore, the predictive value of our identified clusters remains to be established, underscoring the need for longitudinal follow-up of our patient cohort to validate these results over time.

Finally, the semi-quantitative analysis of blood flow patterns and manual 2D analysis plane positioning introduce a limitation due to potential observer-dependent inaccuracies and the comparative lack of precision against more advanced, automated techniques.

## Conclusion

Hierarchical cluster analysis based on aortic 4D flow MRI and Z-score revealed three distinct subgroups of Marfan patients, each characterized by specific hemodynamic profiles and clinical features. Follow-up of our patients is warranted to assess if 4D flow MRI- and Z-score-based stratification can predict future aortic diameter growth and ultimately improve outcomes.

## Supplementary information

ELECTRONIC SUPPLEMENTARY MATERIAL

movie_cluster1

movie_cluster2

movie_cluster3

## Abbreviations

2D: Two dimensional
4D: Four dimensional (time-resolved 3-dimensional)
AA: Ascending aorta
MFS: Marfan syndrome
midAA: Mid-ascending aorta
proxAA: Proximal aortic arch
STJ: Sinotubular junction
