# Supplementary material for: Cluster analysis of 100 Marfan patients based on aortic 4D flow MRI and Z-score: insights into disease heterogeneity and stratification of subgroups
Source: Eur Radiol. 2024 Sep 16;35(4):2200–12. doi: 10.1007/s00330-024-11034-6 (PMC11913908; doi:10.1007/s00330-024-11034-6)
Supplement: Supplementary file 1 — ELECTRONIC SUPPLEMENTARY MATERIAL [file 330_2024_11034_MOESM1_ESM.pdf]

Cluster analysis of 100 Marfan patients based on aortic 4D flow MRI and Z-score: Insights into disease heterogeneity and stratification of subgroups
ELECTRONIC SUPPLEMENTARY MATERIAL

movie_cluster1 (MPEG-4, m4v) Physiological blood flow in a 41-year-old female Marfan patient from cluster 1 with a Z-score of 0.3.

movie_cluster2 (MPEG-4, m4v) Moderate *helical* (grade 1) blood flow in a 20-year-old female Marfan patient from cluster 2 with a Z-score of 3.4.

movie_cluster3 (MPEG-4, m4v) Pronounced combined *helical* (grade 2) and *vortical* (grade 2) blood flow in a 60-year-old female Marfan patient from cluster 3 with a Z-score of 4.0.
